# A69 ASSESSING COVID-19 VACCINATION COMPLIANCE AND HESITANCY IN THE EARLY ADULT (18-25YRS) INFLAMMATORY BOWEL DISEASE (IBD) POPULATION

**DOI:** 10.1093/jcag/gwac036.069

**Published:** 2023-03-07

**Authors:** A Ramkissoon, Z Mohmand, N Bollegala

**Affiliations:** 1 Women's College Hospital; 2 University of Toronto, Toronto, Canada

## Abstract

**Background:**

Effective strategies at educating high risk IBD patients about COVID-19 and the benefits of vaccination is critical.

**Purpose:**

The primary objective of this study was to assess the impact of early adulthood (18-25 years) on COVID-19 vaccination compliance. Secondary objectives were to assess the impact of early adulthood on vaccine hesitancy and reasons for non-compliance.

**Method:**

A retrospective chart review was performed on all eligible IBD patients. Inclusion criteria: patients followed at Women’s College Hospital, age 18 years and older, diagnosis of Crohn’s disease (CD), Ulcerative Colitis (UC) or Indeterminant Colitis (IC). Data was collected between January 01 and August 31, 2022. Demographic data, level of immunosuppression and COVID-19 vaccine status were collected. All participants identified as not fully vaccinated (defined as less than 3 COVID-19 vaccines) were enrolled into the second phase of the study in which a modified Oxford COVID-19 Vaccine Hesitancy Scale was administered, and reasons for vaccine hesitancy discussed and addressed using a prepared educational template. Chi-square test evaluated the impact of age on vaccination status. Adjusted logistic regression was also performed. Fisher exact test evaluated the impact of age on willingness to accept vaccination counseling. Generalized linear regression model evaluated the impact of this on vaccine hesitancy.

**Result(s):**

287 patients were included in the study of which 254 (89%) were immunosuppressed, 197 (69%) were female. 144 (50%) had CD, 136 (47%) had UC and 7 (2.4%) had IC. There were significantly more young adults who were under/unvaccinated 22/82 (27%) compared to older adults 22/205 (11%), p=0.0006 . Gender, IBD phenotype, immunosuppression status did not significantly affect vaccination status. The only variable found to significantly affect vaccination status on adjusted logistic regression was age, >25 years associated with OR 3.25 (95% CI 1.63-6.50) for full vaccination. 32/39 study subjects who were un/under-vaccinated agreed to counseling (82% of the early adults and 82% of older adults). Gender, IBD phenotype, immunesuppression status and age had no impact on uptake. Linear regression modeling found no significant impact on vaccine hesitancy by age, gender, IBD phenotype or immunesuppression. The main reasons described by patients for hesitancy included safety concerns and potential side effects (43%), perceived low risk (27%), prior COVID-19 infection (17%), lack of knowledge of current public health guidance (13%).

**Conclusion(s):**

Early adult IBD patients were found to be significantly less compliant with obtaining COVID-19 vaccines. This hesitancy mainly stems from concerns regarding vaccine safety and efficacy and perceived low risk. More targeted education strategies are required to increase awareness and ensure vaccine compliance.

**Please acknowledge all funding agencies by checking the applicable boxes below:**

None

**Disclosure of Interest:**

None Declared

